# High-Speed and Accurate Diagnosis of Gastrointestinal Disease: Learning on Endoscopy Images Using Lightweight Transformer with Local Feature Attention

**DOI:** 10.3390/bioengineering10121416

**Published:** 2023-12-13

**Authors:** Shibin Wu, Ruxin Zhang, Jiayi Yan, Chengquan Li, Qicai Liu, Liyang Wang, Haoqian Wang

**Affiliations:** 1 Shenzhen International Graduate School, Tsinghua University, Shenzhen 518055, China; wusb20@mails.tsinghua.edu.cn (S.W.); zrx18@mails.tsinghua.edu.cn (R.Z.); yanjy21@mails.tsinghua.edu.cn (J.Y.); 2 School of Clinical Medicine, Tsinghua University, Beijing 100084, China; licq19@mails.tsinghua.edu.cn; 3 Vanke School of Public Health, Tsinghua University, Beijing 100084, China; liuqc22@mails.tsinghua.edu.cn

**Keywords:** gastrointestinal disease, lightweight, fast, accurate, transformer-based model

## Abstract

In response to the pressing need for robust disease diagnosis from gastrointestinal tract (GIT) endoscopic images, we proposed FLATer, a fast, lightweight, and highly accurate transformer-based model. FLATer consists of a residual block, a vision transformer module, and a spatial attention block, which concurrently focuses on local features and global attention. It can leverage the capabilities of both convolutional neural networks (CNNs) and vision transformers (ViT). We decomposed the classification of endoscopic images into two subtasks: a binary classification to discern between normal and pathological images and a further multi-class classification to categorize images into specific diseases, namely ulcerative colitis, polyps, and esophagitis. FLATer has exhibited exceptional prowess in these tasks, achieving 96.4% accuracy in binary classification and 99.7% accuracy in ternary classification, surpassing most existing models. Notably, FLATer could maintain impressive performance when trained from scratch, underscoring its robustness. In addition to the high precision, FLATer boasted remarkable efficiency, reaching a notable throughput of 16.4k images per second, which positions FLATer as a compelling candidate for rapid disease identification in clinical practice.

## 1. Introduction

Gastrointestinal tract (GIT) disease is a global health concern due to its high prevalence and impact on mortality rates, causing millions of health-related cases and deaths annually [1,2]. The most common incidents include stomach, colorectal, and esophageal cancers [3]. Despite alarming statistics, gastrointestinal disorders are frequently overlooked and underdiagnosed. This results in a substantial disparity in detection rates for various GIT diseases, for example, with around 20% of polyps being missed undergoing gastrointestinal endoscopy [4]. Among the various gastrointestinal diseases, some occur frequently, such as ulcerative colitis, polyps, and esophagitis. Ulcerative colitis is a chronic condition affecting the large intestine, characterized by inflammation and ulcers of the colon’s inner lining [5]. Polyps are abnormal tissue growths that can occur in organs like the stomach and colon [6], potentially developing into colon cancer over time. Esophagitis refers to inflammation of the esophagus, often caused by acid reflux [7].

Diagnosis of these diseases mainly relies on endoscopic examination, which involves inserting a flexible tube into the patient’s body to visualize the GIT [8]. However, this procedure is often painful and uncomfortable for patients. Wireless Capsule Endoscopy (WCE) has significantly improved this process, allowing direct visualization of the GIT by having patients swallow a small camera capsule [9,10,11,12]. WCE has benefited around 1 million patients in 2018 with less invasive and more comfortable procedure [13]. Despite these advancements, interpreting endoscopic images remains challenging, even for experienced professionals, as some diseases’ images can be misleadingly similar [14]. For instance, expert endoscopists can accurately identify high-grade neoplasia via gastroscopy, while novices achieve only 69% sensitivity in detection [15]. Therefore, there is a critical need for a swift, accurate, and automated diagnosis method to assist in detecting and classifying gastrointestinal diseases. Rapid development and emerging application of artificial intelligence (AI), particularly deep learning (DL), in the domain of medical imaging diagnosis has garnered substantial attention. Among DL models, Convolutional Neural Networks (CNNs) have shown promise in diverse applications, including GIT lesion analysis, and they are usually superior to traditional methods [16,17,18].

Disease diagnosis based on medical images can often be translated into typical computer vision tasks, including segmentation, classification, detection, and so on. CNNs use an end-to-end architecture to extract local features, which contain a lot of information about points and edges, color, and texture from input images [19]. With adequate training and sufficient data, CNNs can potentially outperform humans in the above-mentioned vision task. Focusing on GIT disease classification from WCE images, previous works have achieved great performance based on the CNN framework. Majid et al. [20] proposed an ensemble classifier for gastric infection recognition via WCE using a VGG16-based CNN framework, including a fusion of features, which has achieved 96.5% accuracy. Komeda et al. [21] developed a ResNet-based AI model for diagnosing colorectal polyps, utilizing a large dataset of polyp images and demonstrating high accuracy and diagnostic value. ECA-DDCNN [22] employed efficient channel attention and deep dense convolutions and accurately classified esophageal gastroscopic images into four categories and seven sub-categories of esophageal diseases. Yogapriya et al. [23] solved the classification problem by a hybrid approach of adjusted pre-trained CNN models, with VGG16 achieving the highest results. Wang et al. [24] presented a novel residual learning method with deep feature extraction for diagnosing celiac disease through an analysis of video capsule endoscopy images, showing significant potential. However, CNN-based models face challenges in capturing global characteristics and long-range dependencies, which are crucial for disease recognition precision in endoscopic images [25]. The inherent similarity in shapes and textures among different GIT diseases, along with blurred lesions and normal tissue distinctions, limits CNN-based approaches, hampering further improvements.

Recently, the transformer has revolutionized deep learning, utilizing attention mechanisms for improved interpretability and performance in various domains, including natural language processing and computer vision [26]. Vision Transformers (ViT), which employ self-attention to capture global correlations among image patches, have been applied to GIT disease diagnosis using WCE images, achieving state-of-the-art results [27,28]. However, ViT outperforms CNNs mainly with ample data for pre-training, and their computational demands can hinder efficiency, as observed in predicting esophageal variceal bleeding [29].

**Figure 1:**
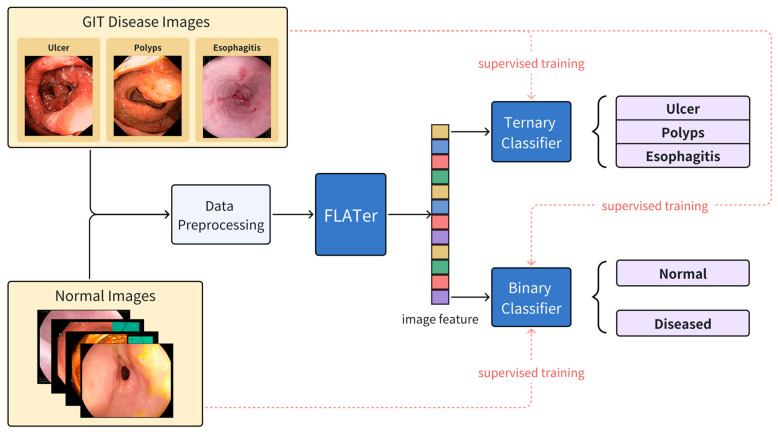
The workflow of the proposed FLATer for GIT disease detection and classification.

In this paper, we proposed a Fast, Lightweight, and Accurate Transformer-based model (FLATer) for GIT endoscopic image disease classification. FLATer combines the strengths of CNNs and transformers, utilizing residual convolutional blocks to extract local features and introducing spatial attention for improved adaptability to limited datasets. To address the prevalence of healthy regions in GIT endoscopic images, we augmented the “normal” label in the dataset, ensuring a substantial proportion of normal data. FLATer incorporates both binary and ternary classifiers to identify lesion areas and classify specific diseases, streamlining the disease detection and classification process. We conducted a thorough ablation study to validate each module’s effectiveness in FLATer and demonstrated superior performance even without pre-trained parameters. Additionally, FLATer offers a more lightweight alternative to most ViT models, maintaining comparable performance while reducing inference time significantly. The workflow of this study is shown in Figure 1.

## 2. Materials and Methods

### 2.1. Data Collection and Preprocessing

In order to validate the effectiveness of our model, we utilized datasets from the study by Montalbo [30], which includes two publicly available gastrointestinal endoscopy datasets: KVASIR [31] and ETIS-Larib Polyp DB [32]. Gathered through WCE, KVASIR comprises GI images with verified labels from Vestre Viken Health Trust (Norway). All data in KVASIR and ETIS-Larib Polyp DB have elaborate labels to ensure suitability for deep learning research. The datasets consist of normal images and diseased images from three categories: ulcerative colitis, polyps, and esophagitis. Each category had 1500 images, totaling 6000 images.

In addition to the aforementioned datasets, Kvasir-V2 [33] expands upon the original KVASIR dataset, encompassing eight distinct categories: normal-cecum, normal-pylorus, normal-z-line, esophagitis, ulcerative colitis, polyps, dyed lifted polyps, and dyed resection margins. Each category contained 1000 images. Within Kvasir-V2, we merged the ulcerative colitis, polyps, and esophagitis images into the original dataset [30] and removed duplicates. The data from normal-cecum, normal-pylorus, and normal-z-line was consolidated into Kvasir-V1’s normal class, significantly augmenting the volume of normal images. This equalizes the number of healthy images with those of the three disease categories, aligning more closely with the typical distribution in endoscopic imaging under real scenes. As a result, our image classification can be divided into two sub-tasks: a binary classification determines if the image region is normal, and simultaneously, a ternary classification identifies the specific disease category for non-normal images. The data distribution for the binary classification achieves balance across the two categories. In total, we curated approximately 10,000 gastrointestinal endoscopic images, with details on data samples and the division into training, validation, and test sets provided in Table 1.

We conducted preprocessing on the original images of collected datasets. The images in the raw dataset had resolutions ranging from 720 × 576 to 1920 × 1072. For the training set, we applied operations including resize, random resized crop, random horizontal flip, and normalize. For the test set, images underwent resizing, centered crop, and normalization. In normalizing the images, we applied empirical parameters. The mean values for the RGB channels were set to 0.485, 0.456, and 0.406, respectively, with corresponding variances of 0.229, 0.224, and 0.225. Consistent normalization procedures were employed across the training, validation, and test sets. All images input into the network were set to the resolution of 224 × 224.

### 2.2. Model Architecture

#### 2.2.1. Vision Transformer

The Vision Transformer (ViT) [27] adapts transformer mechanism, traditionally applied in natural language processing, to the domain of visual data and was originally designed to solve image classification problems. Given an input image with height H, width W, and C channels, it should be divided into some fixed-size, non-overlapping patches. The patch size P can vary based on the application. In the case of the ViT-B/16 model, 16 × 16 sized patches are used. Each patch is then linearly embedded into a vector whose dimension is dim⁡=P×P×C, by a trainable embedding matrix. The number of image patches can be calculated as $(H\times W)/P^{2}$. These embedded vectors are treated as token sequences, analogous to the word or phrase tokens in text transformer. A “class” token (CLS) needs to be added at the start of the token sequence for image classification. The total length of the token sequence transformed from the input image is $L=(H\times W)/P^{2}+1$. To overcome transformer’s lack of inherent sensitivity to input data order, position embeddings (PE) are used to provide spatial context for image patches. These learnable embeddings are added to patch embeddings, allowing the model to recognize patch spatial arrangements. Multi-head self-attention (MHSA) layers further enhance the model’s ability to focus on important image regions, with the final image representation obtained from the CLS token and used for classification.

#### 2.2.2. Proposed Model—FLATer

**Figure 2:**
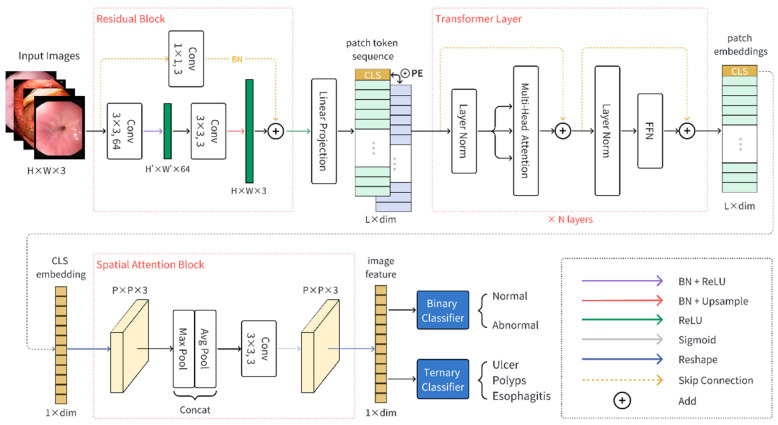
The model architecture of the proposed FLATer.

The proposed FLATer consists of a residual block, a ViT backbone, and a spatial attention block. Overall, the architecture of FLATer is shown in Figure 2. Given a GIT endoscopic image $I\in R^{H\times W\times C}$, we use the residual block to extract the local features from the input image. The residual block introduces inductive bias; such prior knowledge lowers the threshold of the subsequent training of VIT architecture. The image features are divided into image patches and mapped through the linear projection operation to form flattened embedding sequences with $R^{L\times dim}$. Afterward, these embeddings pass through several transformer layers, enhanced with positional information. Specifically, we can load the VIT-B/16 model as initial parameters for transformer layers, which is pre-trained with the large-scared ImageNet-21k dataset. We utilize the output CLS token embedding as the overall representation of the input image. Additionally, we leverage the spatial attention block on the CLS embedding to further enhance the spatial information from a global representation. Finally, the classification head, which consists of a binary classifier and a ternary classifier, gives the probability prediction of preconfigured normal or disease category labels.

The residual block is a single encoder–decoder layer, which consists of three 3 × 3 convolution layers. The first convolution operation is followed with batch normalization and ReLU activation function, which can be formulated as (1).

(1) $$z_{1}=ReLU(BN({Conv}^{1}(I)))$$

We set the stride of the first convolution layer to be greater than 1, which means it can be considered as down-sampling. The second convolution block is followed with a batch norm layer and up-sample operation. The sequential procedure is formulated as (2).

(2) $$z_{2}=US(BN({Conv}^{2}(z_{1})))$$

where US denotes the bilinear interpolation function for up-sampling. Besides, the input image is processed with a residual connection, which is made up of a 1 × 1 convolution block and a batch norm layer, and then is added to $Z_{2}$.

(3) $$F_{1}={ReLU(z}_{2}+BN({Conv}^{3}(I)))$$

Transformations between the number of feature channels are indicated in Figure 2. The output feature $F_{1}$ of a residual block is converted into a patch token sequence and propagates forward through N transformer layers. As described in Section 2.2.1, the vision transform module can be formulated as follows.

(4) $$f_{0}=\left[CE,TE^{1},TE^{2},\ldots ,TE^{L}\right]+PE$$

(5) $$f_{i}^{\prime }=MHSA\left(LN\left(f_{i-1}\right)\right)+f_{i-1}$$

(6) $$f_{i}=FFN\left(LN\left(f_{i}^{\prime }\right)\right)+f_{i}^{\prime }$$

where $f_{i}$ denotes the output feature of the *i*-th transformer layer and $f_{i}^{\prime }$ denotes the intermediate feature. PE indicates position embeddings, while CE refers to the CLS embedding. Each image patch $E^{i}$ is projected to a flattened vector by a linear mapping matrix T.

We denote the CLS vector of the last transformer layer’s output as $F_{2}$, which can be considered as a global representation of the input image. After the propagation of several attention layers, we need to recognize the local information of the output feature again. To that end, we designed a spatial attention module after the transformer block. Firstly, the one-dimensional embedding $F_{2}$ should be reshaped into a patch feature map $F_{2}^{\prime }$ with P×P×C dimension. The following max pooling and average pooling are leveraged in extracting relevant spatial information from the feature map. Subsequently, the results from the max pooling and average pooling are concatenated along the channel dimension, followed by a 3 × 3 convolution layer. The final feature map is reshaped back to a one-dimensional feature F. The spatial attention module can be formulated as follows.

(7) $$F^{\prime }=Conv\left(\left[MaxPool\left(F_{2}^{\prime }\right),\;AvgPool\left(F_{2}^{\prime }\right)\right]\right)$$

(8) $$F=reshape\left(Sigmoid\left(F^{\prime }\right)\right)$$

The spatial attention mechanism effectively modulates the importance of different regions within the input feature, emphasizing regions of significance while suppressing irrelevant or noisy information. The final feature is used for classification.

#### 2.2.3. Comparison Models

In our study, we employ abundant comparison models to benchmark the performance of FLATer with common standards. GoogLeNet [34] introduced the inception module, optimizing computational cost while increasing the depth and width of neural networks. DenseNet [35] employs dense connections between layers, enhancing gradient flow and feature reuse. ResNet [36], particularly the 18-layer or 50-layer variants, utilizes skip connections to combat vanishing gradients in deep networks. ResNeXt50 [37] extends ResNet by introducing grouped convolutions, increasing model capacity without added complexity. VGG [38] is characterized by its simplicity, using only 3 × 3 convolutional layers stacked on top of each other in increasing depth. EfficientNet [39] systematically scales the network width, depth, and resolution for improved performance. MobileNet [40] employs depth-wise separable convolutions, significantly reducing the number of parameters and catering to mobile and embedded applications. Xception [41], inspired by Inception, replaces inception modules with depth-wise separable convolutions. The majority of the aforementioned comparative models utilize CNN architectures. Similarly, the foundational architecture of ViT [27], devoid of auxiliary components, serves as a baseline for comparison. In the experimental setup involving loaded pre-trained parameters, all aforementioned models underwent pre-training on the ImageNet dataset or its subsets [42]. ImageNet dataset is known to contain over 14 million labeled images spanning approximately 21,000 object categories. It is one of the largest and most widely used natural image datasets for training and evaluating computer vision models.

### 2.3. Experimental Set Up

In our experiment, we used GIT endoscopic image datasets categorized into normal and diseased, training a binary classifier on these categories. Within the diseased category, we further divided the images into three specific disease types (ulcerative colitis, polyps, and esophagitis) to train a ternary classifier. Both binary and ternary classification tasks were trained simultaneously, sharing core network parameters with differences only in the final classification layer. In scenarios where all models are initialized with pre-trained parameters, we conducted training and comparative analyses involving FLATer and other models. Additionally, we performed an ablation study to systematically evaluate the effectiveness of FLATer’s individual sub-modules. We also evaluated FLATer’s performance against ResNeXt50 and ViT models, all without pre-trained parameters. Additionally, we examined FLATer’s ViT block with varying encoder layer configurations (12, 6, 4) to investigate the impact of model size reduction. All experiments were performed on an NVIDIA GeForce RTX 3090 GPU with 24GB VRAM and a system equipped with a 32-core CPU and 64GB RAM, running Ubuntu 18.04 and Python 3.8.13 with the PyTorch 1.12.1 deep learning framework.

### 2.4. Model Evaluation

In medical imaging, the assessment of diagnostic performance for GIT diseases relies on the examination of a confusion matrix, encompassing key elements including True Positives (TP), False Positives (FP), True Negatives (TN), and False Negatives (FN). The classification performance of the proposed model is comprehensively evaluated through five metrics based on the confusion matrix. These metrics include accuracy (11), precision (9), recall (10), F1-score (12), and AUC [43]. In the ternary classification, we employ the macro-average calculation method to compile statistics across multiple disease class labels. These general evaluation indicators are defined as:

(9) $$precision=\frac{TP}{TP+FP}$$

(10) $$recall=\frac{TP}{TP+FN}$$

(11) $$accuracy=\frac{TP+TN}{TP+FP+TN+FN}$$

(12) $$F1\_score=2\frac{precicion\times recall}{precision+recall}$$

The AUC reflects the area under the ROC curve. The ROC curve is generated by plotting the True Positive Rate (TPR), also known as sensitivity, against the False Positive Rate (FPR), which is calculated as 1 minus specificity at various threshold values. Each point on the ROC curve represents the model’s performance at a specific threshold for classifying data points.

## 3. Results

### 3.1. Binary Classification Results

We categorized the dataset into normal and disease images, training a binary classifier to effectively distinguish between them, specifically identifying abnormal images with lesions in gastrointestinal endoscopic images. We conducted a comprehensive comparison between FLATer and various widely used classification models, including DenseNet121, EfficientNetB0, MobileNetV2, ResNet18, Resnet50, ResNeXt50, VGG16, Xception, and GoogLeNet. FLATer consistently outperformed these models, achieving an accuracy score of 96.63%, a precision score of 96.40%, a recall score of 98.07%, and an F1 score of 97.21% on the test dataset, highlighting its strong generalization capability. The results in Table 2 demonstrate the superior performance of FLATer in comparison to these counterparts.

**Figure 3:**
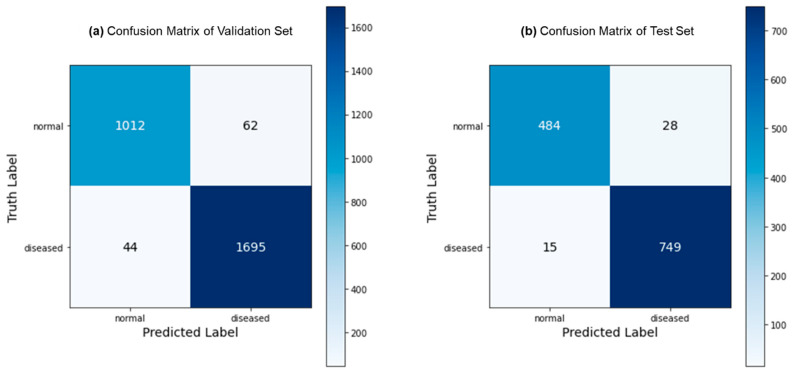
Confusion matrix for the binary classification: validation (**a**) and test (**b**).

**Figure 4:**
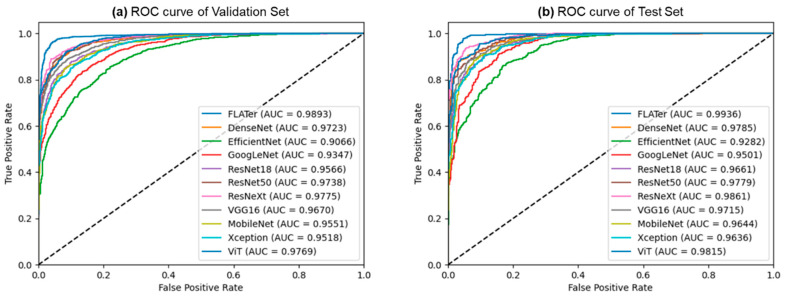
The comparison results of ROC curves from the validation (**a**) and test (**b**) datasets.

Figure 3 depicts the predictions generated by the proposed FLATer model, as illustrated through confusion matrices, utilizing both the validation and test datasets. The model’s performance is notably commendable, with only 106 misclassified samples out of 2813 within the validation dataset and 43 misclassified samples out of 1276 within the test dataset. Figure 4 presents the ROC curves for FLATer and the comparative methods, reflecting their respective performances on the validation and test datasets. Remarkably, our proposed model achieves an impressive AUC of 98.93% on the validation dataset and 99.36% on the test dataset, surpassing the performance of the comparative methods in terms of AUC.

The generalization capability of FLATer is investigated through a progressive dataset extension strategy. This approach involves the consolidation of the validation and test datasets, resulting in a merged dataset comprising 4089 samples. Specifically, training is conducted using the entire training dataset, which consists of 5645 samples. Subsequently, the model’s performance is assessed when utilizing 20%, 40%, 60%, 80%, and 100% of the samples from the merged dataset. The results in Table 3 demonstrate that FLATer exhibits robust inference performance, achieving an accuracy of 96.36%, a precision of 96.45%, a recall of 97.64%, and an F1 score of 97.04% at a 100% sample rate of the merged dataset. Moreover, it is noteworthy that FLATer’s performance remains stable across datasets of varying scales, consistently yielding an F1 score exceeding 97%.

### 3.2. Ternary Classification Results

After effective identification of abnormal images by the binary classifier, the multi-classifier can predict the specific disease type corresponding to the abnormal images. In our experiments, a ternary classifier was utilized for category prediction on several typical GIT diseases, including ulcerative colitis, polyps, and esophagitis. The ternary classification comparison result is shown in Table 4. Our proposed model attains an accuracy score of 99.77%, a precision score of 99.78%, a recall score of 99.78%, and an F1 score of 99.78% on the validation dataset. Likewise, on the test dataset, FLATer achieves an accuracy score of 99.61%, a precision score of 99.60%, a recall score of 99.61%, and an F1 score of 99.60%. This near-100% performance on ternary classification highlights the significant capabilities of FLATer in handling the disease classification problem of GIT endoscopic images.

**Figure 5:**
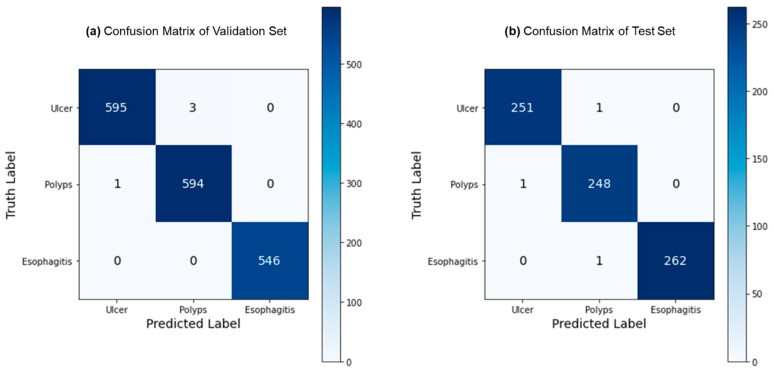
Confusion matrix for the ternary classification: validation (**a**) and test (**b**).

**Figure 6:**
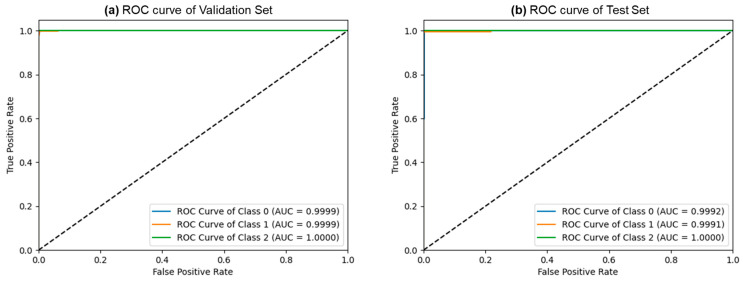
The ROC curves of the ternary classification from the validation (**a**) and test (**b**) sets.

Figure 5 depicts the confusion matrices for the ternary classification of the validation and test datasets. Remarkably, the validation dataset exhibited only 4 misclassified samples out of 1144, while the test dataset had just 3 misclassified samples out of 764. This exceptional performance indicates FLATer’s superiority in ternary classification compared to its binary classification results. Figure 6 presents the ROC curves for FLATer in ternary classification, demonstrating its performance on the validation and test datasets. Classes 0, 1, and 2 correspond to GIT image datasets for ulcerative colitis, polyps, and esophagitis. Notably, the graph shows an average AUC of 1.00 for both datasets, confirming the model’s outstanding capability to handle diagnostic tasks effectively across various thresholds. This consistent performance on both datasets underscores the model’s robustness and reliability, regardless of the types of diseases considered.

We also execute the progressive dataset extension strategy in the ternary classification. The results, detailed in Table 5, reflect FLATer’s remarkable generalization capabilities. Notably, all performance indicators exceed 99%, underscoring the model’s robustness and capacity to generalize effectively.

In conclusion, the pre-trained transformer model can acquire rich prior knowledge during pre-training on large-scale datasets. It enables ViT to grasp a comprehensive understanding of general image representations compared to the CNN-based model. When fine-tuned for a specific task, such as endoscopic image classification, the pre-trained ViT module included in FLATer not only accelerates the model’s convergence but also significantly enhances performance.

### 3.3. Ablation Study Results

**Figure 7:**
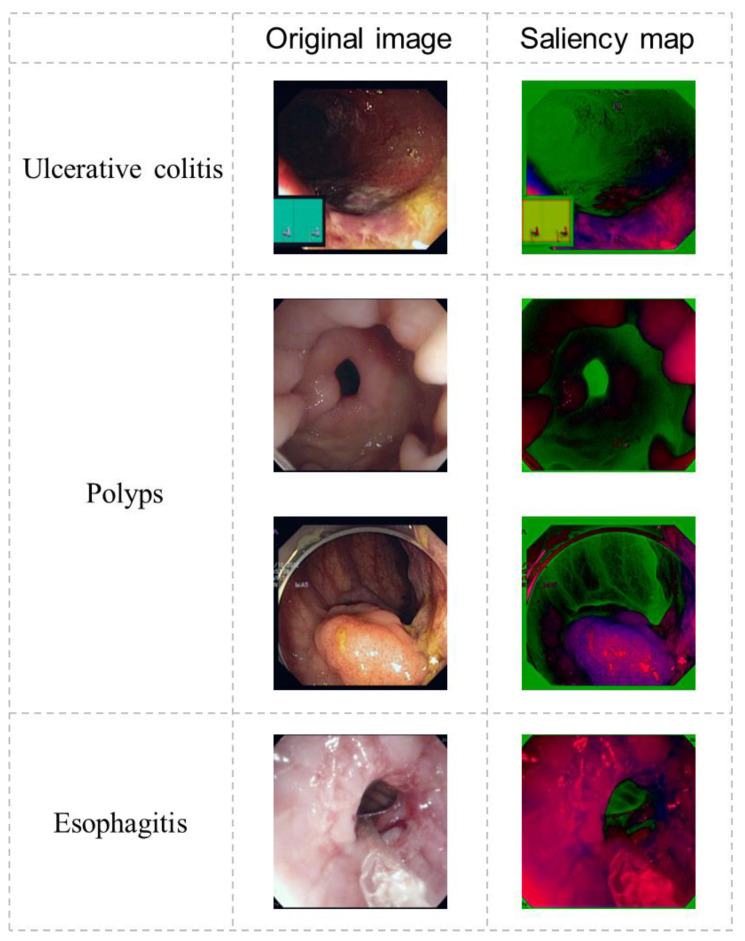
The output saliency maps of the residual block.

We conducted a thorough ablation study to assess each module’s effectiveness in FLATer, examining variants of the model without specific components. The experiments aimed to emphasize FLATer’s architectural design and the contributions of the additional residual block and spatial attention module in enhancing accuracy and robustness for GIT disease classification. Table 6 presents the results of our ablation study, where we removed the residual block and spatial attention modules and even used the ViT model without additional structures. The findings revealed that removing these modules from FLATer resulted in varying degrees of reduced classification accuracy for both binary and ternary classification, with the original ViT model experiencing the most significant performance degradation. Notably, the performance drop was smaller when removing only the spatial attention compared to removing only the residual block. In the test set of ternary classification, it achieved results identical to the full FLATer structure (99.6% accuracy), indicating its robustness with minor struggles against outliers. This underscores the critical role of a CNN-based local feature extraction network in enhancing model accuracy. Additionally, Figure 7 illustrates the saliency maps of diseased samples after passing through the residual block, demonstrating CNN’s ability to highlight pathological regions.

## 4. Discussion

In this work, we aim to tackle the challenge of accurate disease classification of GIT endoscopic images under the condition that the data distribution is closer to the real scene. The proposed FLATer leverages the capabilities of both CNNs and ViT. It concurrently extracts local features while establishing the global correlation. In extensive experiments, FLATer has achieved 96.23% accuracy in binary classification and 99.77% in ternary classification, outperforming various pre-trained CNN models and ViT. The ablation study underscored the effectiveness of each FLATer’s module in reaching the optimal results of disease classification on gastrointestinal endoscopic images.

In our experiments, we tested FLATer, a CNN-based model (ResNeXt50-32x4d), and ViT with randomly initialized model parameters, demonstrating FLATer’s adaptability without pre-training. FLATer consistently outperformed all metrics in binary and ternary classification across validation and test datasets (as shown in Table 7). For binary classification, FLATer achieved 91.72% accuracy on the validation set and 92.24% on the test set, while for ternary classification, it reached 95.86% on the validation set and 96.60% on the test set. ResNeXt50 had slightly lower performance, while ViT exhibited a significant drop in performance when trained from scratch. This highlights the transformer’s lack of inductive bias compared to CNNs, impacting its performance with limited training data. When training on a relatively limited dataset with randomly initialized parameters, such as endoscopic images, the CNN module within FLATer introduces an inductive bias. Simultaneously, the inclusion of the attention block proves instrumental in capturing long-range dependencies and establishing global correlations among the local features. The synergistic collaboration between CNNs and transformers in FLATer thus ensures robust performance across various scenarios, demonstrating the model’s efficacy in both pre-trained and scratch-trained settings.

**Figure 8:**
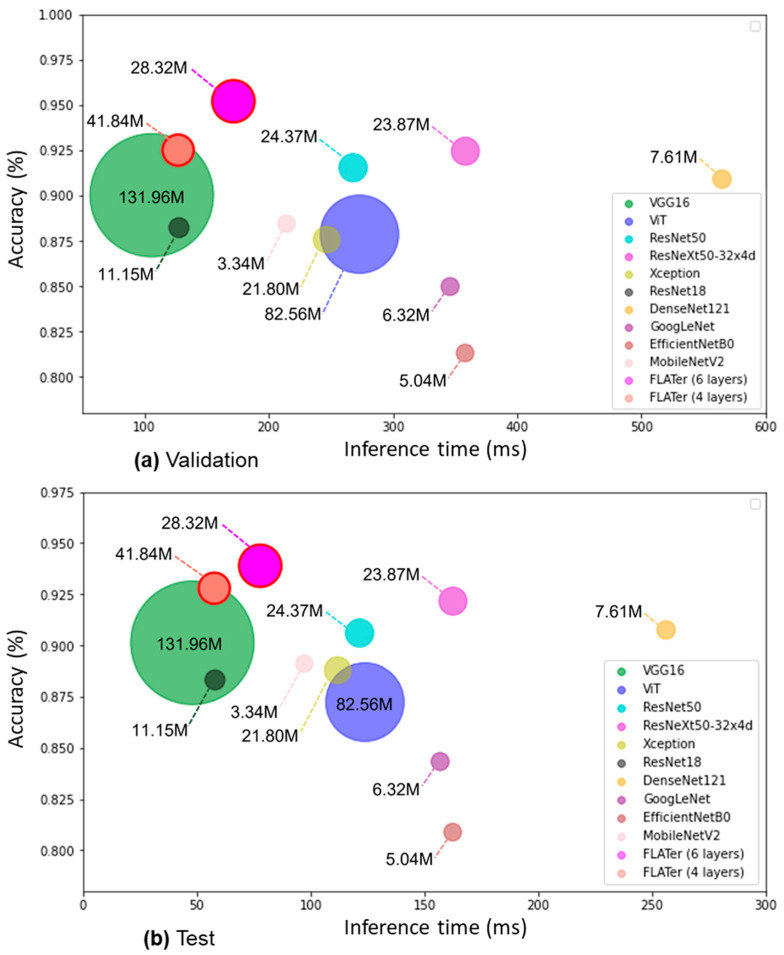
Comparison of model’s averaged inference time to accuracy ratios in binary classification: validation (**a**) and test (**b**).

We conducted experiments with varying numbers of transformer layers in FLATer, as shown in Table 8. Reducing the number of layers from 12 (standard ViT-B/16 [27]) to 6 resulted in comparable classification performance for GIT endoscopic image classification, making FLATer efficient for faster model inference. However, further reducing the number of layers to 4 led to a more significant decline in classification performance. Efficiency comparisons in Figure 8 demonstrate that the 6-layer FLATer maintained high accuracy while significantly reducing inference time. For binary classification with 2813 validation samples and 1276 test samples, the 6-layer FLATer achieved a total inference time of 249 ms, while the 4-layer model took 185 ms. The lightweight FLATer achieved an impressive throughput of 16.4k images per second, slightly below VGG16 and ResNet18. However, VGG16 and ResNet18 experienced accuracy drops compared to FLATer. Furthermore, Figure 8 displays the total number of trainable parameters for each model, highlighting FLATer’s fewer parameters compared to VGG16 and ViT, making it comparable to ultra-lightweight CNN models. These results underscore FLATer’s capability for rapid and accurate disease classification in real medical scenarios.

There are several limitations to our proposed method. Firstly, our dataset primarily includes only three gastrointestinal diseases. Future research should prioritize the collection of data encompassing a broader spectrum of gastrointestinal diseases to enhance the model’s diagnostic capabilities. Secondly, our study lacks external independent testing, and further validation of the model’s generalizability should be conducted using non-public datasets from multiple medical centers. Additionally, our study predominantly focuses on the classification problem, and future research should explore the integration of segmentation and other common medical image visualization tasks to expand our model’s functionality. Finally, we have not developed a deployable software system with a user-friendly graphical interface. We will consider developing such a system to facilitate its clinical application.

## 5. Conclusions

In this study, we introduced FLATer, a transformer-based model designed for accurate and efficient GIT disease diagnosis using endoscopic images. FLATer leverages both CNNs and transformer structures to deliver exceptional performance. Our experiments demonstrate that FLATer outperforms a variety of comparison models while maintaining efficiency in inference times. The results underscore the clinical potential of FLATer, offering rapid and precise disease type prediction suitable for real-world medical applications. Future work will involve expanding the dataset to include a broader range of gastrointestinal diseases, conducting external independent testing with non-public datasets, exploring additional medical imaging tasks like segmentation, and developing a user-friendly software system for practical clinical deployment.

## Figures and Tables

**Table 1:** Dataset specifications.

Samples	Class	Train	Validation	Test	Total
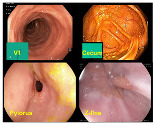	Normal (V1/cecum/ pylorus/z-line)	2800	1074	512	4386
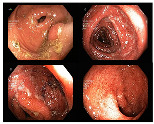	Ulcerative Colitis	948	598	252	1798
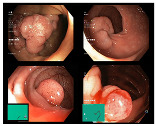	Polyps	949	595	249	1793
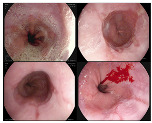	Esophagitis	948	546	263	1757
	Total	5645	2813	1276	9734

**Table 2:** The binary classification comparison results between FLATer and other models from the validation (left) and test (right) sets.

Model	Validation Set				Test Set			
	Accuracy	Precision	Recall	F1-Score	Accuracy	Precision	Recall	F1-Score
FLATer	0.9623	0.9647	0.9747	0.9697	0.9663	0.9640	0.9804	0.9721
ViT-B/16	0.9206	0.9139	0.9212	0.9175	0.9169	0.9434	0.9162	0.9296
DenseNet 121	0.9090	0.9473	0.8580	0.9004	0.9075	0.9792	0.8639	0.9179
EfficientNetB0	0.8130	0.8425	0.7504	0.7938	0.8088	0.9075	0.7579	0.8260
MobileNet V2	0.8844	0.9044	0.8488	0.8757	0.8911	0.9471	0.8665	0.9050
ResNet18	0.8822	0.9044	0.8436	0.8730	0.8832	0.9489	0.8508	0.8972
ResNet50	0.9153	0.9197	0.9022	0.9109	0.9060	0.9435	0.8966	0.9195
ResNeXt50-32x4d	0.9244	0.9475	0.8919	0.9188	0.9216	0.9729	0.8940	0.9318
VGG16	0.9001	0.9106	0.8781	0.8940	0.9013	0.9443	0.8874	0.9150
Xception	0.8756	0.8710	0.8695	0.8702	0.8879	0.9355	0.8730	0.9032
GoogLeNet	0.8497	0.8803	0.7947	0.8353	0.8433	0.9338	0.7945	0.8586

**Table 3:** The evaluation results of binary classification on the progressive dataset extension.

Sample Rate	Quantity	Accuracy	Precision	Recall	F1-Score
20%	817	0.9633	0.9607	0.9800	0.9702
40%	1635	0.9645	0.9600	0.9830	0.9713
60%	2453	0.9645	0.9670	0.9753	0.9711
80%	3271	0.9633	0.9649	0.9755	0.9702
100%	4089	0.9636	0.9645	0.9764	0.9704

**Table 4:** The ternary classification comparison results between FLATer and other models from the validation (left) and test (right) sets.

Model	Validation Set				Test Set			
	Accuracy	Precision	Recall	F1-Score	Accuracy	Precision	Recall	F1-Score
FLATer	0.9977	0.9978	0.9978	0.9978	0.9961	0.9960	0.9961	0.9960
ViT-B/16	0.9839	0.9841	0.9844	0.9842	0.9895	0.9894	0.9894	0.9894
DenseNet121	0.9448	0.9596	0.8803	0.9182	0.9594	0.9782	0.9032	0.9392
EfficientNetB0	0.8712	0.8199	0.8440	0.8318	0.9045	0.8846	0.8554	0.8697
MobileNetV2	0.9344	0.9618	0.8499	0.9024	0.9503	0.9692	0.8871	0.9263
ResNet18	0.9103	0.9316	0.8112	0.8673	0.9215	0.9130	0.8537	0.8824
ResNet50	0.9339	0.9635	0.8535	0.9051	0.9398	0.9554	0.8629	0.9068
ResNeXt50-32x4d	0.9500	0.9474	0.9091	0.9278	0.9503	0.9489	0.8956	0.9215
VGG16	0.9097	0.9720	0.7635	0.8553	0.9149	0.9697	0.7773	0.8629
Xception	0.9563	0.9645	0.9172	0.9403	0.9516	0.9496	0.9150	0.9320
GoogLeNet	0.9086	0.8602	0.8910	0.8753	0.9346	0.9032	0.9106	0.9069

**Table 5:** The evaluation results of ternary classification on the progressive dataset extension.

Sample Rate	Quantity	Accuracy	Precision	Recall	F1-Score
20%	499	0.9980	0.9980	0.9979	0.9980
40%	1000	0.9960	0.9961	0.9960	0.9960
60%	1501	0.9967	0.9967	0.9967	0.9967
80%	2002	0.9970	0.9970	0.9970	0.9970
100%	2503	0.9972	0.9972	0.9972	0.9972

**Table 6:** Ablation results on the validation and test sets.

Task	Model	Validation Set				Test Set			
		Accuracy	Precision	Recall	F1-Score	Accuracy	Precision	Recall	F1-Score
Binary Classification	FLATer	0.9623	0.9647	0.9747	0.9697	0.9663	0.9640	0.9804	0.9721
	w/o residual block	0.9366	0.9309	0.9373	0.9341	0.9350	0.9583	0.9319	0.9449
	w/o spatial attention	0.9470	0.9469	0.9425	0.9447	0.9491	0.9641	0.9503	0.9572
	ViT backbone	0.9206	0.9139	0.9212	0.9175	0.9169	0.9434	0.9162	0.9296
Ternary Classification	FLATer	0.9977	0.9978	0.9978	0.9978	0.9961	0.9960	0.9961	0.9960
	w/o residual block	0.9885	0.9889	0.9888	0.9888	0.9869	0.9870	0.9866	0.9867
	w/o spatial attention	0.9914	0.9916	0.9915	0.9916	0.9961	0.9960	0.9961	0.9960
	ViT backbone	0.9839	0.9841	0.9844	0.9842	0.9895	0.9894	0.9894	0.9894

**Table 7:** The comparison results of FLATer and several typical models without pre-training.

Task	Model	Validation Set				Test Set			
		Accuracy	Precision	Recall	F1-Score	Accuracy	Precision	Recall	F1-Score
Binary Classification	FLATer	0.9172	0.9363	0.9293	0.9328	0.9224	0.9427	0.9267	0.9347
	ResNeXt50	0.9068	0.9354	0.8654	0.8990	0.8934	0.9591	0.8586	0.9061
	ViT	0.8786	0.8887	0.8539	0.8710	0.8723	0.9238	0.8573	0.8893
Ternary Classification	FLATer	0.9586	0.9602	0.9597	0.9597	0.9660	0.9655	0.9654	0.9654
	ResNeXt50	0.9356	0.9584	0.8550	0.9037	0.9450	0.9602	0.8750	0.9156
	ViT	0.8752	0.8534	0.7757	0.8127	0.8835	0.8419	0.7912	0.8157

**Table 8:** The comparison results of FLATer with different number of transformer layers.

Task	Model	Validation Set				Test Set			
		Accuracy	Precision	Recall	F1-Score	Accuracy	Precision	Recall	F1-Score
Binary Classification	12-layers	0.9623	0.9647	0.9747	0.9697	0.9663	0.9640	0.9804	0.9721
	6-layers	0.9520	0.9718	0.9500	0.9607	0.9389	0.9610	0.9359	0.9483
	4-layers	0.9250	0.9494	0.9281	0.9386	0.9279	0.9565	0.9215	0.9387
Ternary Classification	12-layers	0.9977	0.9978	0.9978	0.9978	0.9961	0.9960	0.9961	0.9960
	6-layers	0.9896	0.9902	0.9899	0.9899	0.9948	0.9948	0.9946	0.9947
	4-layers	0.9724	0.9742	0.9731	0.9731	0.9817	0.9822	0.9813	0.9814

## Data Availability

The model presented in this study and the released code is available at https://github.com/bisawsb/FLATer (accessed on 4 December 2023).
